# Healthy Eating in the Australian Coal Mining Industry: Assessing the Efficacy of the ‘Out of the Box’ Workplace Health Promotion Program

**DOI:** 10.3390/nu15143254

**Published:** 2023-07-22

**Authors:** Aaron Bezzina, Lee Ashton, Trent Watson, Carole L. James

**Affiliations:** 1 Centre for Resources Health and Safety, College of Health, Medicine and Wellbeing, University of Newcastle, Callaghan, NSW 2308, Australia; 2 School of Health Sciences, College of Health, Medicine and Wellbeing, University of Newcastle, Callaghan, NSW 2308, Australia; lee.ashton@newcastle.edu.au (L.A.); twatson@ethoshealth.com.au (T.W.); 3 School of Education, College of Human and Social Futures, University of Newcastle, Callaghan, NSW 2308, Australia; 4 Active Living Research Program, Hunter Medical Research Institute (HMRI), Lot 1 Kookaburra Circuit, New Lambton Heights, NSW 2305, Australia; 5 Ethos Health, Newcastle West, NSW 2302, Australia; 6 Sydney School of Health Sciences, Faculty of Medicine and Health, The University of Sydney, Camperdown, NSW 2006, Australia; carole.james@sydney.edu.au

**Keywords:** coal mining, nutrition, fruit, vegetable, knowledge, health promotion, occupational health

## Abstract

Noncommunicable diseases are the world’s leading cause of death. To curb the global rise in these diseases, using the workplace as a front to disseminate health communication messages and resources has been suggested. This study aimed to assess the efficacy of a workplace health promotion program, ‘Out of the Box’, that targeted nutrition outcomes and nutrition guideline knowledge. A 6-month workplace health promotion program was implemented within a coal mine site. Over the 6 months, there were four wellness focus areas, lasting 1 month each, including fruits, vegetables and portion controlling, label reading, alcohol awareness, and hydration promotion. The study utilized a quasi-experimental pre-test–post-test design, with measurements via self-reported paper-based surveys. At baseline there were 163 responses, and 106 at follow-up. At the 6-month follow-up, respondents had increased odds of recalling the current fruit (OR 1.29, *p* = 0.032) and vegetable (OR 1.76, *p* < 0.001) guidelines. Being male was associated with lower vegetable intake (B: −0.28), although this did not reach statistical significance. A nutrition-focused workplace health promotion program can be an efficacious strategy in improving knowledge of fruit and vegetable guidelines. Further research is needed to evaluate the effectiveness of these programs over time.

## 1. Introduction

The mortality rate attributable to noncommunicable disease (NCDs) has dramatically increased in recent decades. Reports from the World Health Organization highlight that in 2022, 74% of deaths globally resulted from NCDs, rising from 63% in 2013 [1,2]. There are several risk factors that propagate the global burden of these diseases, including genetic, environment, and sociodemographic factors. Notwithstanding, most approaches that look to mitigate these diseases are centred around modifying behavioural aspects which compound the morbidity and mortality of NCDs, including tobacco use, physical inactivity, unhealthy diet, and the harmful use of alcohol [3,4,5]. The most prevalent NCD is cardiovascular disease (CVD), accounting for an estimated 17.9 million deaths each year [6]. In addition to increasing mortality, disability-adjusted life years for CVD have also risen by 16.4% between the years 2007 and 2017 [7], further compounding the need for action.

Diet can have a profound effect on health. Current evidence suggests that several dietary components are important modifiable risk factors for the prevention of a number of chronic diseases [8]. Such chronic diseases include CVD, certain cancers, type 2 diabetes, and illnesses associated with obesity and mental health [9,10]. The Global Burden of Disease study highlights that in 2017, more than 11 million diet-related deaths occurred. These deaths could be linked to a handful of dietary risk factors, including a high intake of sodium (3 million), a low intake of whole grains (3 million), and a low intake of fruits (2 million) [11]. Dose–response meta-analytic prospective cohort studies have further underscored this message. In 2014, Wang et al. [12] highlighted in their review of 833,234 participants across 16 different prospective cohort studies that eating more fruit and vegetables was associated with a lower risk of cardiovascular mortality (hazard ratio of 0.96, *p* = 0.02). Aune et al. [13] further reinforced this in 2017, estimating that insufficient fruit and vegetable intake (below 500 g/day and up to 800 g/day) may be responsible for 5.6 million and 7.8 million premature deaths worldwide [13].

Whilst the link between increased fruit and vegetable consumption and CVD is well established, the ever-ubiquitous nature of CVD and chronic disease presents a treatment challenge for healthcare professionals and policy makers. Novel treatment approaches have been proposed, including using the workplace to disseminate and promote positive health behaviours [14]. There is a growing evidence base to support workplace health promotion programs [14], especially with regard to modifying dietary and obesogenic habits, as well as improving cardiometabolic health outcomes [14]. Specifically, a meta-analysis of 121 studies [14] found that workplace wellness programs can statistically improve fruit and vegetable consumption (0.27 servings per day [95% CI 0.16 to 0.37]), as well as systolic blood pressure (–2.03 mm Hg [–3.16 to –0.89]) and bodyweight (–0.92 kg [–1.11 to –0.72]) [14]. One potential reason for the success of these programs is that workplaces can offer the necessary infrastructure and sustained reach to a large number of individuals for prolonged periods each day [15]. This larger reach also means that workplaces have access to demographics who would otherwise be difficult to engage in health promotion.

Globally, Australia is one of the biggest mining nations [16], and this is just one example of a blue-collar, male-dominated industry that could benefit from workplace health promotion programs. Employees in this industry have also reported a lower consumption of fruits and vegetables compared to gender- and age-matched general population samples [17,18]. In a cross-section of 949 coal miners across New South Wales (NSW), Australia, it has been reported that only 3.5% of employees meet the current recommendations for vegetable intake, and 42.2% for fruit intake (compared to 9% and 51.4% nationally) [17]. The mining industry is a major employer in Australia, hiring more than 250,000 individuals in 2021 [19], and is a significant contributor to the Australian economy, accounting for nearly 10% of Australia’s GDP [20]. Hence, efforts to improve fruit and vegetable consumption amongst this population could have far-reaching positive health benefits for a large employee base within Australia.

There exists a dearth of literature regarding the efficacy of workplace health promotion programs which look to increase nutrition knowledge and outcomes within male-dominated blue-collar industries. This paper presents baseline and follow-up findings from the ‘Out of the Box’ workplace health promotion program, which was implemented at a mine site in rural NSW, Australia. The primary aim of this study was to assess the efficacy of a workplace health promotion program that targeted nutrition outcomes and nutrition guideline knowledge. Secondary aims included assessing what, if any, workplace or demographic outcomes implicated fruit and vegetable intake.

## 2. Materials and Methods

### 2.1. Study Design

This study had a quasi-experimental pre-post study design. The research took a whole-of-site approach, whereby all employees were offered the wellness intervention and were invited to take part in the wellness survey at both baseline and follow-up. Baseline data collection was completed in November 2021, with follow-up surveys completed in June 2022. The formatting of this manuscript is in accordance with the Strengthening the Report of Observational Studies in Epidemiology (STROBE) checklist [21].

### 2.2. Recruitment and Participants

Three NSW coal mining organisations, one open cut, one underground, and one coal supply chain organization, were recruited to participate in the pilot study via a convenience sample of mine sites that expressed interest in participation. The three organisations that expressed initial interest (*n* = 3) were sent an information package that outlined the program content, resources, and themes. Three sites joined the study, from the Hunter and Central West regions of NSW, Australia. Due to the COVID-19 global pandemic, one site withdrew participation from the study, citing operational issues brought about by the pandemic. Another site completed the program, although due to organisational constraints data could not be collected and reported. This present study reports baseline and follow-up outcomes for the one site that was able to complete the study, and in which data were able to be collected and reported.

### 2.3. ‘Out of the Box’ Program and Content

The ‘Out of the Box’ program is a 6-month workplace health promotion program. Program content, intervention strategies, and resources were mapped to Bandura’s Social Cognitive Theory (SCT) with appropriate behaviour change techniques and mechanisms for action (summary in Table 1) [22,23]. The program was developed during the COVID-19 global pandemic and was conceptualized to be COVID safe, with content tailored towards the Australian mining industry. The program was broken into monthly wellness focuses including (Month 1) introduction/awareness; (Month 2) label reading/packing a balanced lunch; (Month 3) fruit and vegetables, and portion control; (Month 4) hydration promotion; (Month 5) alcohol awareness; and (Month 6) summary/program wrap-up.

The program utilized both verbal and visual communication modalities to disseminate health messages. Posters, PowerPoint videos (6–10 slides), pre-shift supervisor health briefings (2–3 min in length), and take-home resources formed the backbone of the program, with each wellness focus having different wellness messages. Resources and health messages were thematically gender-targeted towards males due to the male-dominated nature of the mining industry. This included masculine colour schemes in resource design, specifically dark navy blue. The readability of resources and health messages were set to a Flesch–Kincaid score of 60–70, or an equivalent grade level of year 8–9 at high school.

Supplementary Materials Table S1 provides a detailed outline of the intervention components and the various themes associated with each nutrition focus. In brief, there were three main nutrition focuses underpinning the ‘Out of the Box’ program. The first theme focused on fruit, vegetables, and portions, and looked to educate employees about the Australia dietary guidelines. The key messages being promoted was around the consumption of two servings of fruit and five servings of vegetables, alongside increasing self-efficacy around identifying correct serving sizes for both fruits and vegetables. The second theme was centred around packing a balanced lunch for shifts, and basic food label literacy. Workplace practices associated with coal mining lend themselves to a heavier reliance on pre-packaged foods when working. This is due to several factors, including a lack of refrigeration when underground or operating heavy machinery, hands being covered in dirt and dust, or the inability to prepare food due to time pressures associated with long shifts. Increasing knowledge around how to identify healthier options among pre-packaged foods (via label reading) was the key theme being promoted, with a focus on reducing saturated fats, sugar, and sodium. The last theme emphasized hydration promotion (water) and decreasing sugary drink consumption. Resources and messages educated employees about the amount of sugar in popular drinks and emphasized adequate hydration throughout the day.

Due to COVID-19 restrictions, researchers could not attend the site to help administer the intervention, and hence resources were delivered directly to the site for mine staff members to distribute resources and deliver health messages. PowerPoint videos were played in staff muster areas and the presentation was cycled through on repeat for the duration of the month. During pre-start meetings, at the start of the month, supervisors read from a script developed by the research team outlining the wellness focus and key messages. Both PowerPoint videos and supervisor prestart health messages followed the same year 8–9 readability level and were non-technical in intent and wording. Prior to program commencement, the research team held online discussions with WHS managers, emphasizing the significance of adhering to research protocols. These discussions aimed to underscore the importance of following established guidelines and procedures, thereby maintaining the credibility and reliability of study findings.

### 2.4. Wellness Surveys

Paper-based baseline surveys were completed in November 2021, with follow-up surveys finalized in June 2022. An information statement attached to the survey explained the aims of the project, the voluntary and anonymous nature of the research, and the confidentiality of the data collected. Due to privacy concerns raised by the site, there were no identifying data; hence, it was not possible to link responses from the baseline to the follow-up. The information statement outlined that completion of the survey implied consent. The expected completion time of the survey was 15–20 min.

The survey assessed a range of outcomes including personal characteristics, using a mix of closed and open-ended questions. Personal characteristics included the participants’ age, gender, highest education level, and relationship status.

### 2.5. Current Work Situation

Work situation questions pertained to employment status, usual hours worked in a week, shift-work status, and hours worked per shift. All responses were close ended, excluding the usual hours worked per shift, which invited participants to specify the number of hours when they exceeded 12. Occupation aspects were also assessed, identifying the participants’ employment status, shift work status, hours worked per week, and their employment role. Occupational metrics were all closed questions.

### 2.6. Body Anthropometrics

Body anthropometrics were assessed using self-reported weight in kg and height in cm. Body Mass Index (BMI) was calculated during data analysis from participant responses. Perception of weight status (do you consider yourself an acceptable weight, underweight or overweight) and weight status history over the previous year were also noted.

### 2.7. Dietary Frequency

Dietary intake patterns were assessed via a self-reported dietary questionnaire. Dietary outcomes of interest included fruits, vegetables, sugar-sweetened beverages (SSB), cakes, takeaway meals, and fried potato products. Fruit and vegetable intake patterns were assessed via short form questions, such as ‘how many serves of fruit do you usually eat each day?’, with closed ended responses ranging from: ‘I never eat fruit/vegetables’ to ‘I eat 6 or more serves per day’. These questions were based on the Australian National Health Survey 2017–2018, [18] and were validated for use in a general Australian population [24]. Infographics from the Australian Guide to Healthy Eating depicting correct serving sizes were also shown to support accurate responses.

Similarly, SSB intake, cakes, takeaway meals, and fried potato product consumption frequency were assessed via shortform questions, such as ‘How often do you eat cakes, muffins and scones?’ Closed ended responses ranged from ‘less than once per month’ to ‘more than 7 times per week’. SSB, cakes, takeaway meals, and fried potato products were adapted from the Dietary Questionnaire for Epidemiological Studies (DQES) tool [25], which has been validated for use in Australia [26].

### 2.8. Nutrition Knowledge

Fruit and vegetable knowledge was assessed via an open-ended question asking respondents to recount current Australian Dietary Guidelines for adult men and women (2 serves of fruit and 5 serves of vegetables) [27]. Water knowledge was assessed via a four-choice close-ended response asking respondents to identify the current water requirements for men and women (2.6 L and 2.1 L, 8–10 cups, respectively [27]. One cup equates to roughly 250 millilitres of fluid.

### 2.9. Statistical Analysis

Descriptive statistics were created to summarize the demographic information and outcome measures at baseline and follow-up. Categorical variables were summarized through frequencies and percentages (*n* (%)). Numerical variables were summarized through mean and standard deviation (Mean (SD)) and median and interquartile range (Median (Q1, Q3)). Differences between timepoints were assessed using Fisher’s exact test, Pearson’s Chi-squared test, and Wilcoxon rank sum test.

Various models were developed to assess key outcome measures. (i) Ordinal regression: Nutrition outcomes (fruit servings per day, vegetable servings per day, frequency intake of sugar-sweetened beverages, takeaway, fried potato products, cake, and discretionary food items) and timepoint; (ii) Binary logistic regression: Fruit, vegetable, and water guideline knowledge and timepoint; (iii) Linear regression model assessing fruit and vegetable intake with predictor variables. The ordinal regression models’ linearity was assessed via scatterplots to determine the linear relationship between variables. Independence was assessed via residual plots. Normality was assessed via histograms. Homoscedasticity was assessed via scatterplots of residuals against predicated values. The assessment of assumptions showed no signs of visible violation. Models two and three followed similar quality checks. Multicollinearity was assessed via calculating the variance inflation factor for independent variables.

To account for missing data in models, data were inputted via multiple imputation using chained equations (MICE) under the missing at random (MAR) assumption. Statistical analyses were programmed using R version 4.2.2 [28]. Packages utilized included tidyverse for data manipulation, wrangling, and visualization (tidyverse). Regression models were undertaken using the Cumulative Link Models function (clm), as part of the Fitting Generalized Linear Models package (glm2). Variance inflation factor was assessed via the ‘vif’ function in the Companion to Applied Regression (car) package. Tables were generated using the GTsummary. Due to privacy concerns raised by the site, there were no identifying data and hence it was not possible to link responses from baseline to follow-up.

## 3. Results

Table 2 outlines participant demographics and workplace factors for both baseline and follow-up. There were *n* = 163 responses at baseline and *n* = 106 at follow-up. This study took a whole-of-site approach whereby surveys were made available to all employees (*n* = 499), and the resulting response rates for baseline and follow-up were 33% and 21%, respectively. The majority of employees across both timepoints were male (87% and 88%, respectively). The most-reported age category across both timepoints was 55–64 years of age (36% and 35%, respectively), followed closely by 45–54 years of age (19% and 20%, respectively). A trade or apprenticeship was the most-reported highest level of education obtained at both timepoints (37% and 38%, respectively), in addition to a large proportion of respondents working in trade/maintenance roles (46% and 53%, respectively). Across both timepoints, most employees were permanent full-time workers (91% and 93%, respectively) and did not participate in shift work (64% and 73%, respectively). Most employees worked either 39–45 h per week (baseline: 43%, follow-up: 37%) or 46–56 h per week (baseline: 35%, follow-up: 46%).

Table 3 outlines the results for the ordinal regression model investigating the relationship between key nutrition outcomes and timepoint, controlling for hours worked per week. Regarding the primary outcome, fruit and vegetable intake, there were no changes in the odds of reporting higher consumption at follow-up compared to baseline. The odds of reporting a higher frequency of sugar-sweetened beverage consumption increased from baseline to follow-up (*p* = 0.003), as did discretionary food frequency (*p* < 0.001) and fried potato product frequency and takeaway food frequency (*p* = 0.028).

Regarding the other primary outcomes (nutrition knowledge), there were significant changes between baseline and follow-up for both fruit and vegetable knowledge. Respondents at follow-up had a 29% increased chance of correctly recalling the current fruit guidelines (OR 1.29, 95%CI: 1.02–1.62, *p* = 0.032), and a 76% increased chance of correctly recalling the current vegetable guidelines (OR 1.76, 95%CI: 1.40–2.21, *p* < 0.001). The chance of correctly recalling current hydration recommendations increased, although this was not statistically significant.

Table 4 outlines the results of the linear regression model, which examined the predictors of fruit and vegetable consumption amongst respondents. Servings per day of fruits and vegetables was the outcome variable, whilst predictors included demographic and workplace factors, alongside knowledge of fruit and vegetable guidelines. Overall, results were mixed. Working longer hours (39–45 h per week) was associated with a decrease in fruit and vegetable consumption (B: −0.27, 95% CI: −0.50: −0.05, *p* = 0.018 and B: −0.46, 95% CI: −0.72: −0.21, *p* < 0.001). Highest qualification obtained/education level was one of the strongest predictors for vegetable consumption. There was a positive association between those with university degrees and vegetable intake (B: 0.57, 95% CI: 0.25, 0.88, *p* < 0.001). Juxtaposing, there was a negative association between those with school certificates and vegetable intake (B: −0.47, 95% CI: −0.73, −0.21, *p* < 0.001). There was a negative association between those who identified as male and lower levels of vegetable intake; however, this did not reach statistical significance (B: −0.28, 95% CI: −0.57, 0.02, *p* = 0.052). Interestingly, there was a positive association between those who could correctly recall the dietary guideline for vegetables and an increasing consumption of fruits (B: 0.35, 95% CI: 0.22, 0.48, *p* < 0.001) and vegetables (B: 0.85, 95% CI: 0.70, 1.0, *p* < 0.001). However, this relationship was not mirrored with regard to knowledge of the fruit guidelines, and in fact there was a significant negative association between correctly recalling the fruit guidelines and vegetable intake (B: −0.27, 95% CI: −0.43, −0.12, *p* < 0.001).

## 4. Discussion

The primary aim of this study was to assess the efficacy of a workplace health promotion program which aimed to increase the intake of fruits and vegetables amongst employees, as well as increasing the knowledge of nutrition guidelines. While knowledge of fruit and vegetable guidelines increased between baseline and follow-up, there were no significant increases in fruit or vegetable intake between the timepoints. These results are somewhat in contrast to the current available evidence, which suggests that workplace health promotion programs can significantly increase fruit consumption amongst employees. Peñalvo et al. [14] 2021 systematic review and meta-analysis of 121 RCTs and quasi-experimental studies reinforces this notion. The review found that workplace wellness programs can significantly increase fruit intake by 0.20 servings per day; however, there were no significant reported pooled benefits regarding vegetable intake (0.03 serving per day), which mirrors the results of this study and others [29,30,31].

Possible explanations regarding the statistical differences concerning fruit intake between this study and the aforementioned review include utilizing different study designs. The ‘Out of the Box’ program employed a quasi-experimental pre–post design, as opposed to an RCT, and was chosen for ethical and funding reasons. The lack of control and randomization in this study limited the attribution of observed differences specifically to the intervention. In addition, the implications of gender on dietary patterns may also be a confounding reason that fruit intake did not produce a statistically significant difference. The proportion of males within this study was substantially higher compared to the mean number of males in the review (roughly 88% vs. 49%, respectively). Previous studies within coal mining have underscored the low intake of fruit amongst male employees [17]. This may signal that gender-specific interaction effects are at play, highlighting the challenges of getting males to consume a healthy diet.

Another intervention aspect which was highlighted in Peñalvo et al. [14] review, specifically in the univariable meta-regression analysis, was the relationship between the intervention domain ‘screening’ and increased vegetable consumption (0.2 serves per day, *p* = 0.021). Health screening can involve individual dietary feedback from a healthcare professional, which is a powerful mechanism for increasing vegetable consumption (34, 52). Kuehl, Elliot [32] 2014 RCT, which explored the feasibility of a workplace health promotion program amongst law enforcement employees within the United States, serves as an example of an intervention that was effective and incorporated aspects of screening into design and implementation. Overall, individuals in the intervention group at the 6-month follow-up ate 0.30 servings more of vegetables per day compared to the control group (*p* < 0.005). It would be disingenuous to conclude that the program effect was solely related to the use of health screenings, as the intervention was multicomponent and involved various intervention aspects. Nonetheless, Peñalvo et al. [14] highlighted that screening is typically underutilized in workplace health promotion programs (potentially due to associated costs) [33] and may be a powerful intervention tool in increasing vegetable consumption amongst employees. Modifying an adult’s dietary habits is a challenging task. Because of this, it is imperative to consider how interventions can be designed in a manner which can be the most effective. One example of how this can be achieved is through the appropriate use of behaviour change techniques and behavioural psychology, which the present study employed.

Knowledge of how to correctly perform a behaviour is a powerful behaviour change technique regarding improving the health behaviours of individuals [34]. The present study further ratifies this notion, underscoring the efficacy of appropriately worded and curated health messages and resources in increasing knowledge of fruit and vegetable consumption guidelines. Notably, respondents at follow-up had a 29% increased chance of correctly recalling the current fruit guidelines (*p* = 0.032), and a 76% increased chance of correctly recalling the current vegetable guidelines (*p* = 0.001). The literature suggests that individuals who possess knowledge of the dietary guidelines for fruit and vegetable consumption have a higher likelihood of consuming the recommended daily servings of these food groups compared to those who do not have this knowledge [35,36]. This idea is further supported within this study, whereby respondents who could correctly recall the vegetable guidelines consumed significantly more of these food groups. Interestingly, the increased knowledge of fruit and vegetable guidelines did not translate into any significant increases in the consumption of these foods at follow-up. This may be due to the lack of link responses between timepoints, and the inability to account for individual changes and relationships across the intervention. Notwithstanding, this phenomenon potentially signals a greater underlying issue at hand; the interplay between hegemonic masculine culture and poor fruit and vegetable consumption.

One aspect which was highlighted during the data analysis was the negative direction of the relationship between respondents who identified as male and vegetable intake (B: −0.28). Whilst this relationship did not reach statistical significance in this study, this relationship has been echoed by others [37,38,39]. This is especially relevant when considering that 88–89% of respondents at both timepoints were male, which is in line with the reported current demographic characteristics for the Australian coal mining industry [40]. Hegemonic masculine culture, which is synonymous with blue-collar, heavy-based industries such as mining, has been theorized to influence how males perceive the feminized world of dieting [41]. Through a health behaviour lens, the theory of planned behaviour can help explain why hegemonic masculine workplaces can implicate what males eat [42]. Within group settings, men often face pressure within their own gender to act in a predetermined manner of masculinity, which is underpinned by reckless behaviour such as that of poor diet [43]. Reported gender differences within the theory of planned behaviour constructs (attitudes, subjective norms, and perceived behavioural control) posit that females eat more vegetable because of this reason. Namely, they have more positive attitudes towards vegetables, perceived increased social pressures to consume vegetables, and report greater perceived behavioural control over their vegetable intake compared to men [42]. This notion is then contrasted with male-dominated industries such as construction and mining, in which risky behaviour and poor diet are often quoted as a pinnacle cornerstone of masculinity [44]. These social differences may explain some of the reasons why males consume fewer vegetables than their female counter parts.

Socio-economic status, which is a function of educational attainment, income, and occupation, has been shown to be a significant determinant for vegetable consumption patterns [38,45,46,47]. Large (*n* = 37,762 respondents) multi-country (*n* = 21) cross-sectional studies within Europe have highlighted that educational attainment significantly contributes to vegetable consumption patterns, with those with higher education levels (tertiary education) having significantly higher odds (OR: 1.86, *p* < 0.01) of consuming higher intakes of vegetables (>1 serve per day) compared to those with lower educational attainment (school certificate and lower) [38]. This relationship is further echoed within the present study, whereby those respondents who reported high levels of educational attainment (University Degree and Higher University Degree) ate significantly more vegetables. Within Australia, educational attainment is a significant predictor for an individual’s income [48]. Reduced spending power predicated by one’s education and income has been suggested to negatively affect diet quality, resulting in a lower intake of fruits and vegetables [49]. It is important to understand that this notion may not be applicable to the Australian coal mining industry due to the higher-than-average salaries individuals within this industry earn relative to their level of education. As of November 2022, mining industry employees earned on average AUD 2811.70 per week, AUD 1000 more than the average of all industries combined (AUD 1807.70). Perhaps a more appropriate explanation as to why individuals within this industry have poorer vegetable consumption patterns, relative to education attainment and income, is the relationship between nutrition knowledge, cooking skills, and unhealthy food environments [50]. These three factors are thought to be independent of income and have been theorized to be a product of the socio-economic position of an individual during their childhood, rather than their latter adult years [51,52]. This may be the case within the mining industry, whereby the most widely reported level of educational attainment within this study group was a trade/apprenticeship (37–38% across both timepoints), which through a sociological lens has traditionally (although this is changing) been associated with blue-collar, working-class individuals [53]. It is evident that the relationship between socio-economic status, income, and dietary intake patterns is complex. This study aimed to enhance employees’ knowledge of fruit and vegetable guidelines as a strategy to promote an increased consumption of these foods. Whilst the results of this study around intake patterns were not as fruitful as anticipated, the increasing level of recall around fruit and vegetable guidelines is encouraging. Future research within this population should consider the relationship between cooking skills and efficacy and how this may implicate consumption patterns.

One of the curious findings highlighted during the results from this study was the interplay between time and an increased consumption frequency of sugar-sweetened beverages, takeaways, fried potato products, and cakes/confectionary. Whilst on face value it would appear that the intervention was counter-intuitive and increased the consumption of energy-dense, nutrient-poor foods, within the context of COVID-19 these results are understandable. In mid-October 2021, two weeks prior to the commencement of the intervention (November 2021), the state of New South Wales in Australia, in which the intervention site is located, had just ended a 107-day lockdown. This period was marked by the removal of stay-at-home orders, in which the public could re-emergeand socialize for the first time in months. On top of this factor, seasonally, December and January within Australia are some of the busiest socializing months, due to the desirable (summer) weather, festive season, and school summer holidays. These factors could account for some of the variation in dietary patterns between timepoints [54].

Strengths of our study include an intervention which was underpinned by relevant theories and behaviour change techniques, which has been shown to increase the effect size of outcomes. Furthermore, the ‘Out of the Box’ program took a whole-of-site approach. This approach has several benefits, as implementing a health promotion program in a non-controlled environment allows findings to be more applicable to industry, thus increasing the translation of research into practice. However, this approach is not without its limitations, and the lack of randomization or a control group means that caution is prescribed when interpreting results. Limitations include not knowing the exact level of participation in the wellness intervention, as well as the lack of paired data. Limitations induced by COVID-19 ought to be highlighted, especially the inability to assess the fidelity of the intervention due to the research team not being able to attend the site. Furthermore, overall, the response rate of the survey at both timepoints was in comparison to the number of employees on the site. Whilst all employees were made aware of the intervention and could participate, the level of involvement was unknown. While self-reported measures do induce a level of reporting bias, it was not feasible to use objective measures, and the nutrition tools utilized for analysis have been rigorously validated for Australian populations [24,26].

## 5. Conclusions

This study assessed the efficacy of a theory-based nutrition workplace health promotion program in an open cut coal mine site in NSW, Australia, with the primary aim of increasing fruit and vegetable intake and knowledge of guidelines. While the knowledge of fruit and vegetable guidelines increased between the baseline and follow-up, there was no significant increase in fruit and vegetable intake between the timepoints. The ‘Out of the Box’ program highlights the potential for workplace health promotion programs to improve knowledge of current dietary guidelines. Through a behaviour change lens, improving knowledge of behaviour, as well as improving knowledge around how to correctly perform that behaviour, is a powerful mechanism of action in improving health habits. Further research is needed to explore the feasibility and effectiveness of similar interventions in other workplace settings, among different demographic groups, and to ascertain whether these programs can be scaled in size and reach.

## Figures and Tables

**Table 1:** Intervention components mapped to Social Cognitive Theory constructs, behaviour change techniques, and mechanisms of action.

Intervention Component	Social Cognitive Theory Construct	Behaviour Change Techniques	Mechanisms of Action
Pre-shift supervisor health messages	Social support Behavioural capability Goal setting	Social support Goal setting (behaviour)	Social influences via shift supervisors delivering messages. Provide social support and general encouragement. Adhering to national fruit, vegetable, and hydration guidelines
Posters and videos	Self-efficacy Goal setting Behavioural capability	Instruction on how to perform the behaviour. Goal setting (behaviour) Adding objects to the environment	Knowledge of national fruit, vegetable, and hydration guidelines Adhering to national fruit, vegetable, and hydration guidelines Environmental context/resources in the form of education posters and videos
Wallet label reading card	Self-efficacy Behavioural capability Self-control	Behaviour substitution Instruction on how to perform the behaviour	Behavioural regulation via aiding in balanced food selection at the grocery store Knowledge on how to choose balanced food items at the grocery store
Fruit and vegetable fridge magnet	Self-efficacy Behavioural capability Self-control	Adding objects to the environment Prompts/cues Instruction on how to perform the behaviour.	Knowledge of fruit and vegetable guidelines Knowledge of common serving sizes for fruits and vegetables Environmental context/resources in the form of the fridge magnet Behavioural cueing via the fridge magnet
Hydration promotion water bottle	Self-efficacy Behavioural capability Self-control	Adding objects to the environment Prompts/cues	Knowledge of hydration guidelines Environmental context/resources in the form of the water bottle Behavioural cueing via the water bottle

**Table 2:** Participant demographics, baseline, and follow-up.

Characteristic	Baseline, *N* = 163 ^1^	Follow-Up, *N* = 106 ^1^	*p*-Value ^2^
Occupation role			0.6
Office	18 (12%)	9 (8.9%)	
Trade and maintenance	70 (46%)	54 (53%)	
Production	47 (31%)	25 (25%)	
Engineer	6 (3.9%)	6 (5.9%)	
Management	12 (7.8%)	7 (6.9%)	
Missing	10	5	
Gender			>0.9
Female	14 (8.6%)	8 (7.7%)	
Male	142 (88%)	93 (89%)	
Other	2 (1.2%)	1 (1.0%)	
Prefer not to say	4 (2.5%)	2 (1.9%)	
Missing	1	2	
Age group			>0.9
18–24	19 (12%)	14 (14%)	
25–34	23 (15%)	14 (14%)	
35–44	22 (14%)	15 (15%)	
45–54	29 (19%)	20 (20%)	
55–64	55 (36%)	36 (35%)	
65–74	6 (3.9%)	3 (2.9%)	
Missing	9	4	
Highest qualification			>0.9
No formal qualifications	8 (5.0%)	7 (6.7%)	
School certificate	18 (11%)	15 (14%)	
Higher school certificate	17 (11%)	8 (7.6%)	
Trade/apprenticeship	60 (37%)	40 (38%)	
Certificate/diploma	32 (20%)	19 (18%)	
University degree	15 (9.3%)	9 (8.6%)	
Higher university degree	10 (6.2%)	6 (5.7%)	
Prefer not to say	1 (0.6%)	1 (1.0%)	
Missing	2	1	
Relationship status			0.4
Single	13 (8.0%)	10 (9.5%)	
Married/Defacto	124 (76%)	86 (82%)	
Widowed/Divorced/Separated	21 (13%)	8 (7.6%)	
Prefer not to say	5 (3.1%)	1 (1.0%)	
Missing	0	1	
Hours worked per week			0.037
24–38	25 (15%)	6 (5.8%)	
39–45	70 (43%)	39 (38%)	
46–56	57 (35%)	49 (47%)	
>56	11 (6.7%)	10 (9.6%)	
Missing	0	2	
Shift work status			0.10
Yes	59 (36%)	28 (26%)	
No	104 (64%)	77 (74%)	
Missing	0	1	
Body weight (kg)	91 (16)	91 (17)	0.8
Missing	8	3	
Body Mass Index (kg/m^2^)	28.2 (3.9)	28.4 (4.2)	0.7
Missing	20	11	
Fruit intake			0.8
Serves of fruit per day	1 (0.2)	1 (0.2)	
Missing	4	1	
Vegetable intake			0.8
Serves of vegetables per day	2 (1.3)	2 (1.3)	
Missing	2	1	

^1^ *n* (%); mean (SD); median (IQR). ^2^ Fisher’s exact test; Pearson’s Chi-squared test; Wilcoxon rank sum test.

**Table 3:** Ordinal regression results of primary outcomes: baseline vs. follow-up.

Characteristic	OR ^1^	95% CI ^1^	*p*-Value
Fruit servings per day	1.10	0.90, 1.34	0.4
Vegetable servings per day	0.98	0.80, 1.19	0.8
Sugar-sweetened beverage frequency	1.35	1.11, 1.64	0.003
Cake frequency	1.47	1.20, 1.80	<0.001
Fried potato products frequency	1.32	1.08, 1.61	0.006
Takeaway frequency	1.25	1.03, 1.53	0.028
Fruit guideline knowledge	1.29	1.02, 1.62	0.032
Vegetable guideline knowledge	1.76	1.40, 2.21	<0.001
Water guideline knowledge	1.39	0.94, 2.07	0.10

^1^ OR = odds ratio, CI = confidence interval controlling for hours worked per week.

**Table 4:** Predictor variables for fruit and vegetable intake.

	Fruit Intake (Servings)			Vegetable Intake (Servings)		
Characteristic	Beta	95% CI ^1^	*p*-Value	Beta	95% CI ^1^	*p*-Value
Age group						
18–24	Ref	Ref	Ref	Ref	Ref	Ref
25–34	−0.08	−0.34, 0.17	0.5	0.21	−0.09, 0.50	0.2
35–44	0.05	−0.20, 0.30	0.7	0.38	0.09, 0.66	0.010
45–54	−0.02	−0.26, 0.23	0.9	−0.27	−0.55, 0.02	0.064
55–64	0.32	0.10, 0.53	0.004	0.17	−0.08, 0.42	0.2
65–74	−0.11	−0.48, 0.27	0.6	0.65	0.22, 1.1	0.003
Hours worked per week						
24–38	Ref	Ref	Ref	Ref	Ref	Ref
39–45	−0.27	−0.50, −0.05	0.018	−0.46	−0.72, −0.21	<0.001
46–56	−0.18	−0.41, 0.04	0.11	−0.11	−0.37, 0.15	0.4
>56	−0.65	−0.96, −0.35	<0.001	−0.09	−0.44, 0.26	0.6
Occupation role						
Office	Ref	Ref	Ref	Ref	Ref	Ref
Trade and maintenance	−0.35	−0.59, −0.10	0.006	−0.15	−0.43, 0.13	0.3
Production	−0.28	−0.53, −0.02	0.037	−0.16	−0.46, 0.14	0.3
Engineer	−0.04	−0.41, 0.32	0.8	−0.34	−0.76, 0.08	0.11
Management	−0.27	−0.58, 0.04	0.091	0.20	−0.15, 0.56	0.3
Gender						
Female	Ref	Ref	Ref	Ref	Ref	Ref
Male	0.15	−0.11, 0.40	0.3	−0.28	−0.57, 0.02	0.052
Other	0.13	−0.51, 0.77	0.7	−0.56	−1.3, 0.18	0.14
Prefer not to say	−0.08	−0.57, 0.40	0.7	−1.0	−1.6, −0.48	<0.001
Highest qualification						
Certificate/diploma	Ref	Ref	Ref	Ref	Ref	Ref
Higher school certificate	0.14	−0.12, 0.39	0.3	0.35	0.05, 0.64	0.021
Higher university degree	0.15	−0.16, 0.46	0.3	0.35	−0.01, 0.71	0.052
No formal qualifications	0.16	−0.14, 0.46	0.3	−0.16	−0.51, 0.19	0.4
Prefer not to say	−0.51	−1.3, 0.25	0.2	−2.0	−2.8, −1.1	<0.001
School certificate	−0.22	−0.44, 0.01	0.066	−0.47	−0.73, −0.21	<0.001
Trade/apprenticeship	−0.06	−0.24, 0.11	0.5	−0.41	−0.62, −0.20	<0.001
University degree	−0.08	−0.35, 0.19	0.6	0.57	0.25, 0.88	<0.001
Fruit knowledge						
Incorrect	Ref	Ref	Ref	Ref	Ref	Ref
Correct	−0.05	−0.19, 0.08	0.4	−0.27	−0.43, −0.12	<0.001
Vegetable knowledge						
Incorrect	Ref	Ref	Ref	Ref	Ref	Ref
Correct	0.35	0.22, 0.48	<0.001	0.85	0.70, 1.0	<0.001

^1^ CI = confidence interval. Note: ‘Ref’ denotes the reference category for each independent categorical variable.

## Data Availability

Data are available only upon request. Due to the data management plan approved by the University of Newcastle’s human ethics committee, data were not able to be placed in a public repository. The conditions under which ethical approval was granted preclude the sharing of these data in a repository, as participants have been assured of its storage and use by the research team only. For any requests or inquiries related to data, please contact the University of Newcastle’s human ethics committee (human-ethics@newcastle.edu.au) referencing project H-2019-0087.

## Supplementary Materials

The following supporting information can be downloaded at: https://www.mdpi.com/article/10.3390/nu15143254/s1, Table S1: Intervention components for the individual nutrition focuses.
