# Supplementary material for: Healthy Eating in the Australian Coal Mining Industry: Assessing the Efficacy of the ‘Out of the Box’ Workplace Health Promotion Program
Source: Nutrients. 2023 Jul 22;15(14):3254. doi: 10.3390/nu15143254 (PMC10384585; doi:10.3390/nu15143254)
Supplement: Supplementary file 1 [file nutrients-15-03254-s001.zip › nutrients-2475459-supplementary.pdf]

Supplementary materials

Table S1: Intervention components for the individual nutrition focuses.

Intervention Focus	Poster	Videos	Take home resource	Supervisor briefings
Fruit, vegetables, and portions	**Poster 1: "Simple Swaps for Smarter Snacking".** Depicted swapping a fun size (350 kj) piece of confectionary for a similar energy composition fruit item.<br>**Poster 2: "Veggie Dinner Hack".** Outlined how to make a healthy spaghetti Bolognese containing 3-4 serves of vegetables per serve.<br>**Poster 3: "Serving size guide".** Outlined various serving portions for all food groups as well as daily recommendations.	**Video 1: "Aim for 2 & 5".** Outlined fruit and vegetable guidelines, how fruit and vegetables consumption help prevent NCDs, examples of 1 serve of fruit and vegetables.<br>**Video 2: "Pro's guide to portion controlling".** Outlined 5 tips for portion controlling including smaller dinnerware, using hands as serving guides.	**Fruit and vegetable portion fridge magnet.** Provided guidelines for adult men and women to eat 2 serves of fruit per/d, and 5 + serves of vegetables per/d. Illustrated common serving sizes using like for like images such as 1 serve of fresh fruit equates to 1 handful (depicting a cricket ball in someone's palm).	Key messages reminded employees to "to go for 2 and 5. That's 2 serves of fruit and 5 serves of vegetables".
Balanced lunch and food labels	**Poster 1: "A picture of health".** Promoted packing a balanced lunch for work.<br>**Poster 2: "How to read food labels".** Provided common tips to reading food labels including 100g column, serving size, energy, total fat, saturated fat, sugars, fibre, sodium, and ingredients list.	**Video 1: "A picture of health".** Provided 3 simple tips for backing a balanced lunch box including sandwiches, health snacks (popcorn, fruit), and plenty of water.<br>**Video 2: "5 easy tips to reading food labels".** Depicted the same tips from poster 2 with regards to reading food labels.	**Wallet label reading guide card.** wallet label reading card designed to be used at supermarkets that illustrated how to identify healthy items. Included suggested limits for fat, saturated, sugar, sodium, fibre and encouraged water intake.	Key message advocated for packing a balanced lunch and encouraged taking a label reading wallet card to check food labels.
Sugary drink and hydration promotion	**Poster 1: 'Water".** Outlined how water is sugar, preservative, and colour free.<br>**Poster 2: "How much sugar is in your drink".** Outlined the various teaspoons of sugar in common drinks (i.e., 600ml cola ~ 16 tsp). Poster also contained health message warning of links between sugar consumption and NCDs.	**Video 1: "Time to rethink sugary drink".** Outlined facts regarding the amount of sugar in common SSB, cost of daily consumption of SSB, and the amount of weight maintained with 1 can of soft drink each day (when energy is in surplus. Additionally, hydration promotion and guidelines regarding water intake.	**Hydration promotion water bottle.** 700 ml aluminium water bottle with hydration message "drink 3 of these a day to keep dehydration away" and included guidelines for daily fluid intake, advocating for water consumption.	Key message encouraged employees to "aim for 8-10 cups of water a day to stay hydrated". Employees were also explained that "The average 600ml soft drink has 16 teaspoons of sugar".
